# Nafion-212 Membrane Solvated by Ethylene and Propylene Carbonates as Electrolyte for Lithium Metal Batteries

**DOI:** 10.3390/polym15224340

**Published:** 2023-11-07

**Authors:** Daria Voropaeva, Svetlana Novikova, Irina Stenina, Andrey Yaroslavtsev

**Affiliations:** Kurnakov Institute of General and Inorganic Chemistry of Russian Academy of Sciences, Leninsky Avenue, 31, 119991 Moscow, Russia; voropaeva@igic.ras.ru (D.V.); svetlana_novi@mail.ru (S.N.); stenina@igic.ras.ru (I.S.)

**Keywords:** polymer electrolyte, cation-exchange membrane, lithium metal battery, Nafion, ionic conductivity, single-ion conductor

## Abstract

The use of cation-exchange membranes as electrolytes for lithium metal batteries can prevent the formation of lithium dendrites during extended cycling and guarantee safe battery operation. In our study, the Nafion-212 membrane in lithium form solvated by a mixture of ethylene carbonate and propylene carbonate (EC-PC) was used as an electrolyte in a lithium metal battery with the LiFePO_4_ cathode. The Nafion-212-EC-PC electrolyte is electrochemically stable up to 6 V, indicating its suitability for high-energy density batteries. It has an ionic conductivity of 1.9 × 10^−4^ S/cm at 25 °C and a high lithium transference number. The symmetric Li|Nafion-212-EC-PC|Li cell shows a very low overvoltage of ~0.3 V at a current density of ±0.1 mA/cm^2^. At 25 °C, the LiFePO_4_|Nafion-212-EC-PC|Li battery exhibits a capacity of 141, 136, 125, and 100 mAh/g at 0.1, 0.2, 0.5, and 1C rates, respectively. It maintains a capacity of 120 mAh/g at 0 °C and 0.1C with stable performance for 50 charge/discharge cycles. The mechanism of conductivity and capacity retention at low temperatures is discussed.

## 1. Introduction

Currently, lithium-ion batteries prevail in the field of electrochemical power sources; however, their energy densities approach the upper limit of ~255 Wh/kg [1]. Achieving higher energy densities requires the improvement of battery design and the development of new electrochemical energy sources that are not yet widely used. Lithium metal batteries offer the prospect of achieving high energy densities up to 500 Wh/kg due to the lower potential and higher specific capacity of lithium metal compared with the graphite and silicon anodes [1,2,3]. Several issues arise when designing lithium metal batteries, including the growth of Li dendrites, the flammability of liquid electrolytes, and poor electrochemical battery performance [4,5,6]. A widely accepted explanation for dendrite formation is the space charge theory proposed by Chazalviel [7]. In conventional liquid or polymer/salt electrolytes, not only Li^+^ ions move under the applied electric field but also anions, the transference numbers of which can reach 0.8 [8,9]. For the reason that fixed anions cannot participate in electrochemical processes, they accumulate near one electrode, creating a depletion layer at the opposite electrode and resulting in concentration polarization. This leads to the formation and spreading of dendrites. To prevent the formation of lithium dendrites, single-ion-conducting polymer electrolytes with fixed anion groups can be used [10,11,12,13,14,15].

Perfluorinated sulfonic acid membranes with -SO_3_^−^ groups bonded to a perfluorinated polymer matrix are of great interest due to their outstanding transport properties as well as their excellent chemical, thermal, and electrochemical stability [16,17]. Nafion membrane is a commonly used polymer (ionomer) that consists of a nonpolar polytetrafluoroethylene matrix and a side chain -O-CF_2_-CF(CF_3_)-O-CF_2_-CF_2_-SO_3_Li, which effectively delocalizes negative charge. Nafion membranes solvated by organic polar aprotic solvents, which are commonly used in conventional lithium-ion batteries, exhibit high ionic conductivity (10^−4^ S/cm), high Li^+^ transference numbers (>0.9), and stable battery performance when used as electrolytes [17,18,19,20,21,22,23]. As solvents for the solvation of membranes, it is possible to use various aprotic organic solvents used in liquid electrolytes, such as linear and cyclic carbonates, esters, sulfoxides, etc. Propylene carbonate (PC) is a widely used solvent for lithium metal and lithium-ion batteries due to its wide operating temperature range (−49–240 °C), high dielectric permeability, wide electrochemical stability window, and high solvation ability. However, it cannot be used as an individual or main component for lithium metal batteries due to its interaction with lithium, leading to a decrease in battery capacity [24]. The addition of ethylene carbonate (EC), in addition to increasing ionic conductivity due to its high dielectric constant and low viscosity, makes it possible to avoid the problem of lithium dissolution and create a protective SEI that prevents the interaction of the electrolyte with the electrodes.

There are several papers in the literature devoted to the use of solvated Nafion membranes in lithium metal and lithium-ion batteries. For example, the commercial Nafion-211 membrane exhibited an ionic conductivity of 0.21 mS/cm at 70 °C after being soaked in propylene carbonate. Meanwhile, the discharge capacity of the Li-S battery was found to be 1072.8 mAh/g (per sulfur) at a cycling rate of 0.05C and 895 mAh/g, with 89% capacity retention after 100 cycles at 1C [20]. The electrolyte prepared by casting a 20% Nafion solution in water and lower aliphatic alcohols and solvated with both ethylene carbonate and propylene carbonate exhibited an ionic conductivity of 0.36 mS/cm at 20 °C. Furthermore, its discharge capacity was approximately 80 mAh/g at 0.05C when tested in a cell with the LiFePO_4_ cathode and lithium anode [18]. The properties of the electrolytes obtained mainly depend on the solvent nature and the method of membrane production. Meanwhile, the electrochemical characteristics of battery cells with membrane-based electrolytes are determined not only by their conductivity but also by their thickness, as well as by the composition and method of battery cell formation. It is important to consider these factors to optimize the performance of battery cells. In recent years, there has been a shift to thinner Nafion membranes of <50 μm thick in different electrochemical applications. To the best of our knowledge, the 50 μm thick Nafion-212 membranes have not yet been used as an electrolyte in lithium metal batteries.

Another important problem with lithium metal batteries is their behavior at negative temperatures associated with insufficient ionic conductivity of solid-state electrolytes or freezing of liquid electrolytes. The use of cation-exchange membranes with nanosized pores can help solve this problem. Due to the presence of both nonpolar (hydrophobic) perfluorinated matrix and polar (hydrophilic) terminal functional groups in perfluorinated sulfonic acid membranes, self-organization processes occur in which polar functional groups aggregate and form nanoscale clusters. As a result, solvents in solvated cation-exchange membranes can freeze at lower temperatures than pure solvents [25] due to the so-called nanosize effect.

The aim of this study was to produce and characterize polymer electrolytes based on commercial perfluorinated sulfocationic membranes, namely Nafion-212, solvated by a mixture of ethylene carbonate and propylene carbonate (EC-PC). The lithium conductivity, transference numbers, and electrolyte stability against lithium metal were investigated for the first time. Temperature effects were studied for Nafion-212 membranes swelled in an EC-PC mixture in the temperature range −100–+130 °C by the DSC technique. In addition, battery cells with the Nafion-212-based electrolyte and the LiFePO_4_ cathode and lithium anode were tested, including performance at low temperatures.

## 2. Materials and Methods

The following reagents were used in this work: Nafion-212 membrane (DuPont, Wilmington, DE, USA, equivalent weight = 1100 g/mol, 50 μm thick (for dry membrane)), LiOH·H_2_O (Sigma Life Science, Burlington, MA, USA), ethylene carbonate (Gelon, Linyi, China), propylene carbonate (Acros Organics, Germany), *N*-methyl-*2*-pyrrolidone (Sigma-Aldrich, Darmstadt, Germany), liquid electrolyte containing 1 M LiPF_6_ in ethylene carbonate, dimethyl carbonate, and diethyl carbonate (4:3:3) + vinylene carbonate (Gelon, China), lithium metal (Gelon, China), polyvinylidene fluoride (MW = 1100 kDa, Gelon, China), Super P-Li (Gelon, China).

### 2.1. Membrane Preparation

Before use as an electrolyte, commercial Nafion-212 membranes were conditioned using a standard procedure to remove monomer impurities and solvent residues [26]. Ion-exchange capacity (IEC, mg-eq/g) was determined by acid-base titration. For this purpose, the dry membrane in the H^+^-form after conditioning was kept in 0.5 M NaCl for 24 h under constant stirring. The solution was then titrated with ~0.01 M NaOH. The exact concentration of the sodium hydroxide solution was determined by titration with a 0.1 M HCl solution prepared by dilution of the standard titrant. The equivalence point was determined by changing the color of the universal indicator ZIV-1. IEC values were calculated by Equation (1):

(1) $$IEC=\frac{C_{NaOH}\cdot V_{NaOH}}{m\cdot V_{s}}\cdot 10^{-3}$$

where $C_{NaOH}$ (mol/L) and $V_{NaOH}$ (L) are the concentration and volume of NaOH used for titration, respectively, *m* is the weight of a dry membrane (g), and $V_{s}$ is the volume of NaCl (L).

To convert the membranes to Li^+^ form, they were treated with a 0.1 M LiOH solution under continuous stirring for 48 h. To remove the remaining ions, membranes were washed multiple times using deionized water and then dried under vacuum at 50 °C for 12 h to remove the remaining water.

### 2.2. Preparation of Polymer Electrolyte

To prepare the Nafion-212-EC-PC polymer electrolyte, the Nafion-212 membrane was transferred into an argon-filled glove box with a moisture and oxygen content of <5 ppm (SPECS, Moscow, Russia) and then immersed in a solution containing equal volumes of ethylene carbonate and propylene carbonate (EC-PC) for 24 h.

The degree of membrane solvation was determined by the ratio of solvent molecules to membrane functional groups. The solvated membrane was analyzed using differential scanning calorimetry in aluminum crucibles in a helium atmosphere at temperatures ranging from −100 to +130 °C with a heating rate of 10 °C/min using a NETZSCH STA 449F1 (Netzsch, Selb, Germany).

### 2.3. Study of a Polymer Electrolyte

#### 2.3.1. Ionic Conductivity

The ionic conductivity of the Nafion-212-EC-PC polymer electrolyte was investigated by impedance spectroscopy in the temperature range −20…+50 °C. Measurements were carried out using an AC bridge Elins Z-1500J (Elins, Chernogolovka, Russia) in the frequency range of 2.5 MHz–10 Hz in an Al|membrane|Al CR2032 coin-type cell. A Binder MKF115 climate chamber (Binder GmbH, Tuttlingen, Germany) was used to set the required temperature. The resistivity was determined by the cutoff on the active resistance axis in Nyquist plots in the higher-temperature range (from +30 to +50 °C) or by extrapolation of the semicircle on the active resistance axis using the ZView 4 program at lower temperatures (from −20 to +25 °C). The activation energy of ionic conductivity was calculated using the Arrhenius equation from the slope of the temperature dependence of conductivity.

#### 2.3.2. Lithium Transference Numbers

Li^+^ transference numbers (*T_Li+_*) were estimated according to the Bruce-Vincent method [27] in symmetric coin-type cells Li|Nafion-212-EC-PC|Li using an Elins P-20X8 instrument (Elins, Chernogolovka, Russia). For the experiment, a potential difference of ΔV = 10 mV was applied to the cells, and the current before (*I*_0_) and after *(I_f_)* polarization was measured. The interface impedance was measured before (*R*_0_) and after (*R_f_*) polarization by impedance spectroscopy in the frequency range of 500 kHz to 10 mHz. The T_Li+_ values were calculated using Equation (2):

(2) $$T_{Li+}=\frac{I_{f}(\Delta V-I_{0}R_{0})}{I_{0}(\Delta V-I_{f}R_{f})}$$

#### 2.3.3. Electrochemical Stability Window

The electrochemical stability window of the Nafion-212-EC-PC polymer electrolyte vs. Li/Li^+^ was determined by linear voltammetry using a multichannel potentiostat-galvanostat Elins P-20X8 (Elins, Russia). The membrane sample was placed between a stainless steel electrode (SS, as the working electrode) and a lithium electrode (as the counter and reference electrode) and assembled in a coin-type CR2032 cell. Potential scans were performed from 0.0 to 6.0 V (vs. Li/Li^+^) with a sweep rate of 1.0 mV/s. Cyclic voltammetry was performed in the potential range of 2.5–4.1 V in SS|electrolyte|Li cells with a sweep rate of 1.0 mV/s for 10 cycles.

To evaluate the stability of the polymer electrolyte against lithium metal, galvanostatic cycling was carried out at a current density of 0.1 mA/cm^2^ in a symmetric Li|Nafion-212-EC-PC|Li cell using Elins P-20X8 multichannel potentiostat-galvanostat. The cycle time was 1 h (0.05 mAh/cm^2^). Before experiments, the lithium surface was mechanically cleaned from lithium oxide and lithium carbonate.

#### 2.3.4. Lithium Metal Batteries

To evaluate the possibility of using the Nafion-212-EC-PC polymer electrolyte in lithium metal batteries, we tested a coin-type cell with a positive electrode based on LiFePO_4_@C (LFP) composite prepared by the solvothermal method using sucrose as a carbon source according to the procedure described in Ref. [28]. To prepare the positive electrode, an LFP composite containing 5 wt.% carbon was mixed with carbon black and a 5 wt.% solution of PVDF (MM = 1100 kDa) in *N*-methyl-*2*-pyrrolidone (the ratio of LFP:carbon black:PVDF was 85:10:5). The resulting slurry was homogenized on a magnetic stirrer under vigorous stirring for 2 h, then subjected to ultrasonic treatment with an ultrasonic probe for 10 s. The obtained electrode paste was applied to aluminum foil with a thickness of 9 μm using the doctor-blade technique; the thickness of rolling was 200 μm. The resulting cathode sheets were dried at 90 °C for 1 h, then pressed under 1 ton, followed by additional holding at 120 °C in vacuum for 24 h. Round electrodes of 1.6 cm in diameter (2 cm^2^ area with ~5 mg/cm^2^ loading) were cut from the coated foil. During assembly of electrochemical cells, the cathode material was impregnated with a small amount (~30 μL) of liquid electrolyte containing 1 M LiPF_6_ in EC-DMC-DEC-VC.

Electrochemical testing was carried out in galvanostatic mode at cycling rates of 0.1C (17 mA/g), 0.2C (34 mA/g), 0.5C (85 mA/g), and 1C (170 mA/g) at 25 °C in a potential range of 2.5 to 4.1 V using an Elins P-20X8 multichannel potentiostat-galvanostat and at 0 and −5 °C at a cycling rates of 0.1C and 0.5C. The cell capacity Q (mAh/g) was calculated by Equation (3):

(3) $$Q=\frac{I\cdot t}{3.6\cdot m}$$

where *I*, *t*, and *m* are current (A), time (s), and weight (g) of active cathode material, respectively. The Coulombic efficiency (CE) was calculated using Equation (4):

(4) CE=QdQc×100%

where *Q_d_* and *Q_c_* are the discharge and charge capacities, respectively.

## 3. Results and Discussion

Nafion-212 membrane has an ion-exchange capacity of 0.87 mg-eq/g, as determined by acid-base titration. Solvent sorption during solvation of the Nafion-212 membrane results in an increase in its thickness and weight. After being soaked in the EC-PC solvent mixture, Nafion-212 exhibited a degree of solvation of 7.8 solvent molecules per sulfonic acid group, resulting in a thickness increase from 50 μm to 62 μm.

**Figure 1:**
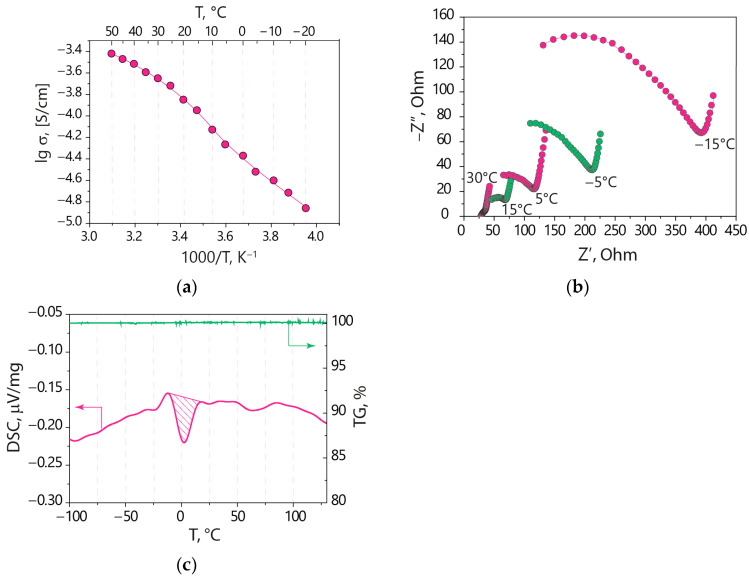
(**a**) Arrhenius plot of ionic conductivity, (**b**) Nyquist plots at different temperatures, and (**c**) weight loss and DSC curves for the Nafion-212-EC-PC polymer electrolyte.

The Nyquist plots and the temperature dependence of the ionic conductivity are shown in Figure 1a,b. At room temperature, the ionic conductivity of the Nafion-212-EC-PC polymer electrolyte is 1.9 × 10^−4^ S/cm, meeting the necessary criteria for electrolytes for lithium metal batteries [29]. The ionic conductivity decreases with decreasing temperature. There are no sharp drops in its temperature dependence; however, its value at −20 °C is an order of magnitude lower than at room temperature.

The DSC curve of the Nafion-212-EC-PC membrane shows a significant endothermic peak with an onset temperature of −5.7 °C (Figure 1c). It can be associated with the melting of a portion of EC in the membrane pores, which is characterized by a higher crystallization temperature [30,31,32]. The absence of abrupt changes in the temperature dependence of conductivity is caused by two factors. In this temperature range, the Nafion-212-EC-PC membrane contains both a liquid EC-PC mixture and solid EC. According to the phase diagram of the binary EC-PC system [32], the eutectic temperature of the EC-PC mixture is about −60 °C for the EC mole fraction of ~0.2. Therefore, above this temperature, the EC-PC mixture contains a liquid phase. With increasing the EC content, the crystallization temperature of EC increases. For the EC mole fraction of 0.56, its melting temperature is 0.9 °C [32]. This is in agreement with our DSC and conductivity data. The difference is that the onset temperature of the endothermic peak observed on the DSC curve in this work is at a slightly lower temperature (−5.7 °C), which may be due to the presence of an impurity (lithium cations) and a decrease in melting temperature in accordance with Raoult’s law. Another reason is that the solvent contains quite a lot of lithium ions, predominantly located within a thin Debye layer near the negatively charged walls of nanosized membrane pores. At these temperatures, only the cation-depleted core (so-called “electrically neutral” solution [25,33]) in solvated nanodomains begins to freeze [34]. As the temperature decreases, a portion of the crystalline solvent with zero cation concentration increases while the concentration of lithium ions in the remaining liquid solvent rises accordingly, which leads to a less sharp drop in the conductivity of solvated membranes than would be in the case of a pure solvent. A similar effect was observed for hydrated perfluorinated sulfonic acid membranes [25] and membranes based on polyethylene and sulfonated grafted polystyrene [34].

**Figure 2:**
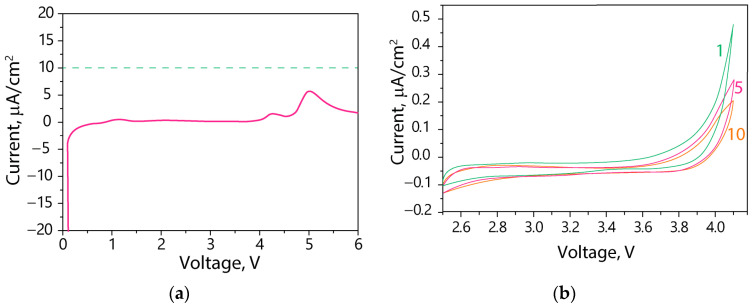
(**a**) Linear (pink line) and (**b**) cyclic voltammetry curves of the SS|Nafion-212-EC-PC|Li cell. Cycle numbers are indicated in the figure.

Based on the linear sweep voltammetry data (Figure 2a), no anodic or markedly cathodic peaks were observed for the Nafion-212-EC-PC electrolyte within the potential range of 0–6 V vs. Li/Li^+^. In fact, the observed cathodic peaks at 4.1 and 5.1 V were characterized by relatively small intensities (<10 μA/cm^2^). Compared with liquid electrolytes stable in the range of ~1–4.5 V [24,35], the Nafion-212-EC-PC electrolyte exhibits a broader electrochemical stability window. Therefore, it can be considered a promising electrolyte compatible with high-voltage cathodes [36,37,38].

Based on cyclic voltammetry in the potential range of 2.5−4.1 V, consistent with the operating range of the LFP cathodes, a gradual decrease in the potential difference between charge-discharge plateaus compared with the first cycle is observed (Figure 2b). This decrease suggests favorable dynamics and increased stability of the electrolyte during cycling.

**Figure 3:**
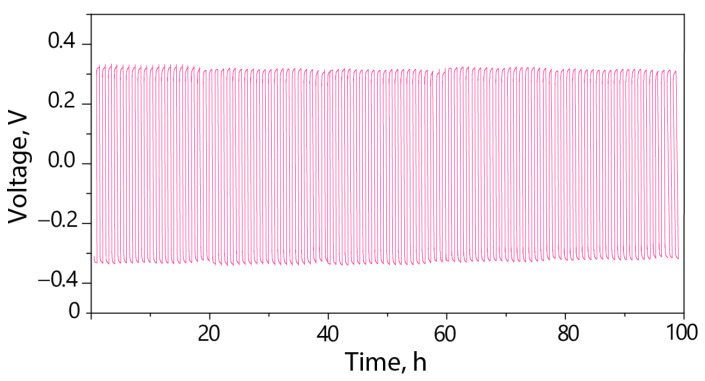
Galvanostatic cycling of Li|Nafion-212-EC-PC|Li cells at 0.1 mA/cm^2^ and cut-off capacity 0.05 mAh/cm^2^.

Based on galvanostatic cycling of a Li|Li cell at a current density of 0.1 mA/cm^2^, the Nafion-212-EC-PC electrolyte exhibits an overvoltage of ~320 mV after 100 cycles (Figure 3). On the voltage profiles, there are no arc-shaped curves that indicate the absence of the polarization effect, which is commonly observed in classical electrolytes with dual-ion conductivity due to the anion concentration gradient [39]. Therefore, the voltage profiles in Figure 3 demonstrate the unipolar conductivity of the Nafion-212-EC-PC electrolyte [40]. The potential values obtained from the galvanostatic cycling plots of Li|Li cells exceed those of certain gel-polymer electrolytes [41,42], which may be due to the slightly lower conductivity of the Nafion-212-EC-PC electrolyte. However, a more likely reason is that batteries with solid electrolytes generally exhibit higher resistances at interfaces between an electrolyte and electrodes as a result of nonoptimal contact.

The Li^+^ transference number is a crucial parameter that determines the performance of an electrolyte in lithium metal batteries. The transference numbers were estimated using the Bruce–Vincent method, which is the most common method for determining Li^+^ transference numbers of polymer electrolytes. It involves the polarization of a symmetric Li|electrolyte|Li cell by a very small (<10 mV) potential difference until the system reaches a steady state with a constant concentration gradient [43]. This method assumes that electrolytes obey the Nernst–Einstein equation, which relates the ion diffusion coefficient to conductivity at infinite dilution and complete dissociation, i.e., an ideal solution without ion-ion interactions. In this regard, values determined by the Bruce–Vincent method and calculated using Equation (2) do not represent the real transference numbers of polymer electrolytes and should be described as a “limiting current fraction”, which refers to the maximum fraction of the initial current that can be maintained without any interfacial resistance in a steady state [44]. There are some other methods for the determination of lithium transference numbers, such as pulsed field gradient NMR [45], electrophoretic NMR [46], the Hittorf [47], and the Newman methods [48]. The disadvantage of these methods is the relatively complex instrumentation required as well as greater measurement errors. Nevertheless, the Bruce–Vincent method is so widely used in the field of polymer electrolytes that it is a standard technique for transference number determination. Therefore, in this work, we used it to compare our data with values reported in the literature.

**Figure 4:**
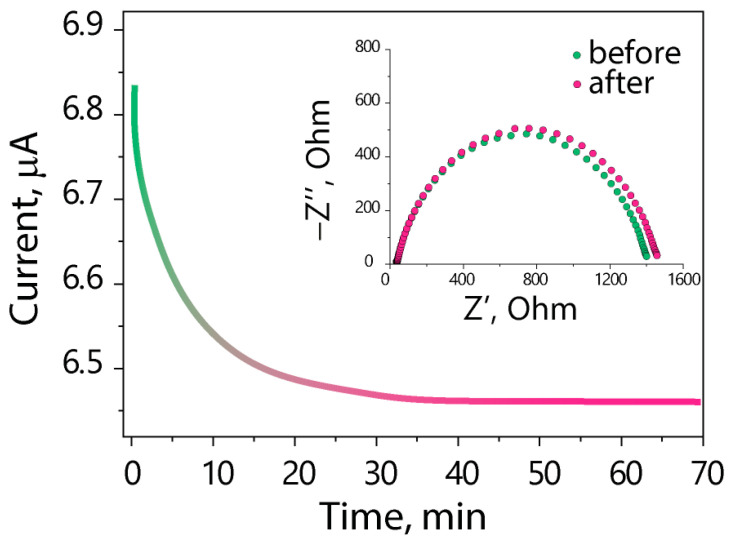
Polarization curve and Nyquist plots of the Li|Nafion-212-EC-PC|Li cell before and after polarization.

Figure 4 shows the Nyquist plots and the polarization curve. The obtained Li transference number for the Nafion-212-EC-PC electrolyte is 0.80, which is comparable to values reported in the literature for single-ion conducting polymer electrolytes, determined by the Bruce–Vincent method [49,50,51]. The difference between the obtained value and 1 may be due to the ion-ion interactions and the segmental mobility of the membrane side chains terminated with SO_3_^−^ groups, which leads to their orientation towards the electrodes under the applied potential difference. Moreover, the concentration of cations at one electrode and cation vacancies at the opposite electrode, which occur because of the polarization (the so-called concentration polarization), also contribute to this difference. The results show that the Nafion-212-EC-PC polymer electrolyte is more selective for cation transfer than liquid electrolytes, which have a transference number of ~0.3 [35].

**Figure 5:**
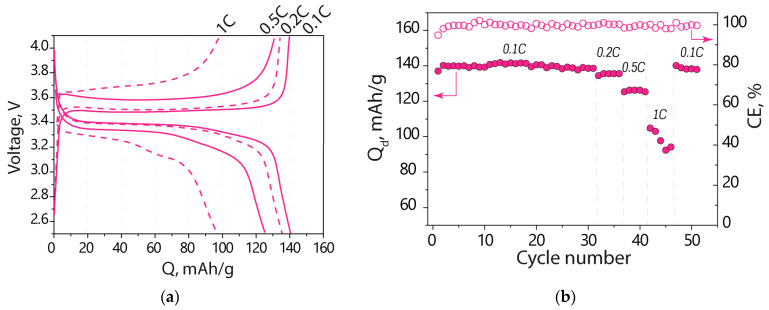
(**a**) Charge–discharge curves at different C-rates at 25 °C and (**b**) the discharge capacity and Coulombic efficiency of the Li|Nafion-212-EC-PC|LFP battery at room temperature and different C-rates (indicated in the figure).

To evaluate the electrochemical performance of a lithium metal battery with an electrolyte based on Nafion-212 solvated by EC-PC, the LFP|Li cell was tested at different charge–discharge rates (Figure 5). Charge–discharge curves of the LFP|Li cells show characteristic plateaus of LiFePO_4_, indicating the Fe^2+^↔Fe^3+^ transition. Meanwhile, the difference between the average potential of charge and discharge plateaus in the cell using the Nafion-212-EC-PC membrane electrolyte is similar to that in cells with liquid electrolytes at the same rates of charge and discharge [28,51], indicating that ohmic losses mainly influence this parameter [52].

The discharge capacity of the Li|Nafion-212-EC-PC|LFP cell was 141 mAh/g at 0.1C, which is less than the capacity of a similar cell with liquid electrolyte by only 11% [28]. This capacity is equivalent to 83% of the theoretical specific capacity of the LFP electrode. As the cycling rate increases, the capacity of the cell with Nafion-212-EC-PC electrolyte decreases to 136, 125, and 100 mAh/g at 0.2, 0.5, and 1C, respectively. The decrease in cell capacity with the Nafion-212-EC-PC membrane electrolyte with increasing C-rate is comparable to that of cells with liquid electrolytes. Increasing the charge/discharge rate by tenfold (up to 1C) results in the cell capacity decreasing by roughly 30%; however, it is recovered when the rate returns to 0.1C (Figure 5b). The recovery of the initial cell capacity indicates the absence of material degradation, and a decrease in the cell capacity at high rates is attributed to kinetic limitations. It should be noted that the Coulombic efficiency of the cell remains around ~100% for all C-rates.

**Figure 6:**
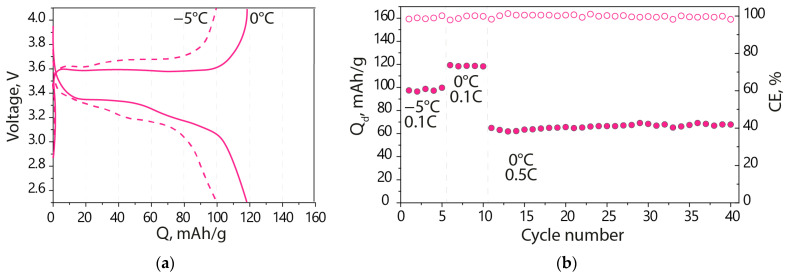
(**a**) Charge-discharge curves at −5, 0 °C and 0.1C and (**b**) the discharge capacity and Coulombic efficiency of the Li|Nafion-212-EC-PC|LFP battery at −5 and 0 °C and 0.1C, 0.5C (indicated in the figure).

Notable is the fact that the cell capacity with the polymer electrolyte remains stable at low temperatures, showing a Coulombic efficiency of ~100%. However, the cell capacity decreased to 100 and 120 mAh/g when cycling at 0.1C at −5 and 0 °C, respectively (Figure 6). This corresponds to a decrease of 29% and 9% in capacity relative to room temperature, despite the fact that the ionic conductivity of the Nafion-212-EC-PC membrane electrolyte is more than an order of magnitude lower at these temperatures. Increasing the cycling rate to 0.5C from 0.1C results in a more significant reduction in capacity, to ~66 mAh/g, which is maintained for 30 cycles with ~100% Coulombic efficiency (Figure 6b). A further temperature decrease leads to a significant decrease in electrochemical capacity due to lower electrolyte conductivity and a slower transfer rate of solvated lithium cation at the electrolyte/SEI interfaces, along with changes in the kinetics of the electrode reaction for LiFePO_4_/FePO_4_ transformation [53,54].

The performance of the Li|Nafion-212-EC-PC|LFP cell at room temperature is comparable with data for other single-ion conducting polymer electrolytes or even surpasses them [18,19,55]. This comparison is also true for cells with liquid electrolytes [51,54,56]. The high-energy density and the ability to operate at low temperatures are among the crucial parameters of modern batteries [57,58,59]. It can be noted that a number of cathode materials are being developed for this purpose [38,60,61,62], while the obstacle to their commercialization is the lack of electrolytes with a wide electrochemical stability window. The obtained results suggest that the Nafion-212-EC-PC membrane may be used as an electrolyte in lithium metal batteries with high-energy-density operating at low temperatures.

## 4. Conclusions

The polymer electrolyte for lithium metal batteries was prepared using a Nafion-212 membrane solvated by a mixture of ethylene carbonate and propylene carbonate. The Nafion-212-EC-PC electrolyte has a high lithium transference number and ionic conductivity of 1.9 × 10^−4^ S/cm at 25 °C. The LiFePO_4_|Nafion-212-EC-PC|Li cell exhibited capacities ranging from 100 to 141 mAh/g at different C-rates (0.1C–1C) at room temperature and a capacity of 120 mAh/g at 0 °C and 0.1C. The battery showed stable performance over 50 charge and discharge cycles with a Coulombic efficiency of ~100%. Moreover, the electrolyte is electrochemically stable up to 6 V, indicating its suitability for high-energy-density batteries.

## Data Availability

Data might be available on request.
